# Case report: a patient with pulmonary arterial hypertension transitioning from a PDE-5 inhibitor to Riociguat

**DOI:** 10.1186/s12890-016-0229-x

**Published:** 2016-05-18

**Authors:** David S. Poch

**Affiliations:** Division of Pulmonary, Critical Care, & Sleep Medicine, University of California, San Diego, CA USA

**Keywords:** Pulmonary arterial hypertension, sGC stimulators, Cyclic guanosine monophosphate, Chronic thromboembolic pulmonary hypertension

## Abstract

**Background:**

We present here the case of a patient with pulmonary arterial hypertension and NYHA Class II symptoms who transitioned from PDE-5i therapy to riociguat. No protocol currently exists for transitioning between these PAH medications.

**Case presentation:**

A 59-year old male with a history of anorexigen use initially presented in 2008 and was felt to have non-operable small vessel disease. His care was transitioned to our center after insurance would not cover high-dose sildenafil in addition to ERA therapy.

**Conclusion:**

This case demonstrates a safe and successful transition from higher dose PDE-5is to riociguat with no interruption in therapy.

## Background

Pulmonary arterial hypertension (PAH) is a rare, life-threatening disease defined by chronically elevated pressure in the pulmonary arteries [1]. The etiology of PAH can be idiopathic, familial, or associated with underlying disease, such as connective tissue diseases, certain autoimmune congenital cardiac conditions, and human immunodeficiency virus. PAH is also caused by exposure to drugs or toxins, namely anorectic agents and other stimulants [2].

The pathophysiology of PAH is multifactorial and includes increased expression of the vasoconstrictors endothelin-1 and thromboxane, along with reduced vasodilator activity of the prostacyclin and nitric oxide (NO) pathways. The resultant pulmonary vascular remodeling produces thickened vessel walls and narrowing of arterial lumens. As the disease progresses, compensatory stress on the right ventricle weakens the cardiac muscle, ultimately resulting in right heart failure [3].

Clinically, PAH is defined as a mean pulmonary arterial pressure (mPAP) of ≥25 mmHg at rest, pulmonary capillary wedge pressure (PCWP) of ≤15 mmHg, and pulmonary vascular resistance (PVR) >3 Wood Units (240 dyn · s/cm^5^) [1]. Patients typically present with dyspnea on exertion (DOE), fatigue, dizziness/syncope, angina, and edema. If untreated or inadequately treated, patients generally have a life expectancy of less than 5 years [4].

Primary treatment goals in PAH are to improve symptoms, stabilize or improve New York Heart Association functional class (NYHA FC), and prevent disease progression. Therapeutic agents act on one of the vasoactive pathways implicated in PAH. Endothelin-receptor antagonists (ERAs), phosphodiesterase-5 inhibitors (PDE-5is), and prostacyclins are mainstays of therapy [5]. Initial therapy choice is dictated largely by WHO FC. The standard treatment approach was, until recently, sequential add-on therapy whereby patients start on one therapy and additional therapies are added until treatment goals are achieved. However, recent data have shown upfront combination therapy to be of benefit and recent guidelines updates from the European Respiratory Society (ERS) and European Society of Cardiology (ESC) provide recommendations for both initial drug combination therapy and sequential drug add-on therapy. Combination therapy is an attractive therapeutic option as it may target all three signaling pathways implicated in the pathogenesis of PAH [5–7].

There are four different classes of compounds approved for PAH, two stimulators (prostanoids and soluble guanylate cyclase [sGC] stimulators) and two inhibitors (ERAs and PDE5 inhibitors). Each of these classes has a distinct molecular target. Riociguat is the first member of a novel class of therapeutics called sGC stimulators [8]. Riociguat has a dual mode of action. It sensitizes sGC to endogenous NO by stabilizing NO–sGC binding. Riociguat also directly stimulates sGC via a different binding site, independently of NO. Riociguat restores the NO–sGC–cyclic guanosine monophosphate (cGMP) pathway and leads to increased generation of cGMP. In PAH clinical trials, riociguat has been shown to significantly improve exercise capacity, as well as improve a range of secondary endpoints, including pulmonary hemodynamics, WHO FC, and time to clinical worsening [9].

## Case presentation

The patient is a 59-year-old man first seen for pulmonary testing and evaluation at the University of California, San Diego (UCSD) Medical Center in 2008. He was referred to our center by his pulmonologist, who suspected PAH based on symptoms of worsening DOE and the patient’s self-reported use of anorexigens in the 1990s.

As part of the initial diagnostic work up in 2008, the patient underwent right heart catheterization (RHC), which indicated an mPAP of 35 mmHg with a PCWP of 6 mmHg; cardiac output (CO) was 6.27 L/min, cardiac index (CI) was 2.8 L/min/m^2^ and PVR was 4.9 Wood Units (388 dyn · s/cm^5^). Vasodilator testing was performed with inhaled nitric oxide and the patient was not vasoreactive.

A ventilation-perfusion (V/Q) scan demonstrated segmental perfusion defects of small to moderate size in both lungs. A pulmonary angiogram revealed narrowing of all pulmonary vessels and distal pruning. The angiogram findings were consistent with a diagnosis of PAH and no findings supporting a diagnosis of chronic thromboembolic pulmonary hypertension (CTEPH) were noted.

At the time of initial diagnosis the patient was NYHA FC III and the etiology of his disease was attributed to a prior history of anorexigen use. His referring pulmonologist initiated first-line therapy with bosentan 125 mg PO BID followed by addition of sildenafil 40 mg PO TID.

In 2011, 3 years after starting first-line therapy, the patient transitioned his care to UCSD. On ECHO, estimated peak PAP had decreased to 27 mmHg. RHC parameters had also improved from baseline evaluation: mPAP was reduced to 22 mmHg, PCWP was similar to initial findings at 5 mmHg, CO had increased to 6.37 L/min, CI was now 2.94 L/min/m [2], and PVR was 3.1 Wood units.

Clinically, the patient was now NYHA FC II and given his positive response to treatment, we made the decision to maintain him on his current PAH regimen of an ERA and PDE-5i. In February of 2014, the patient was seen as part of his routine care at our pulmonary hypertension center with stable NYHA FC II symptoms and a 6MWD of 547 m. Due to a change in insurance coverage the patient was unable to obtain sildenfail 40 mg PO TID and a decision was made to transition him to riociguat.

No protocol currently exists for transitioning patients from a PDE-5i to riociguat. We performed our transition without a washout. The patient took his last dose of sildenafil in the evening and the following morning started riociguat 1 mg TID and titrated up by 0.5 mg every 2 weeks to the maximum therapeutic dosage of 2.5 mg TID. The patient remained on bosentan 125 mg BID. Both the transition to riociguat and the titration to maximum therapeutic dosage were well tolerated. The patient did not report any adverse events during switch or maintenance, with the exception of mild heartburn that was successfully managed with an over-the-counter H_2_ blocker.

An ECHO was performed 3 months after the patient began therapy with riociguat and no significant changes in hemodynamic parameters were noted from the previous study in 2011, when the patient was being treated with a PDE-5i.

Subjectively, the patient reported an improvement in his PAH symptoms on riociguat, especially in the area of exercise tolerance. He stated that while on riociguat he experienced increased energy and less breathlessness, even while performing more strenuous exercise. An amateur cyclist, the patient reports being able to ride his bicycle longer and faster—an important personal goal for him. Repeat 6MWD improved to 606 m. As the patient has remained stable with a subjective improvement in symptoms and an improved 6MWD, he will continue on riociguat 2.5 mg TID. He will undergo further assessments at our center, but we have found both his safe switch to riociguat and his initial therapeutic response to be very encouraging.

## Discussion

Recent clinical guidelines have included riociguat as initial therapy with grade I A/B recommendation for patients with WHO FC II-III PAH [5]. Consensus based guidelines from the American College of Chest Physicians recommended riociguat as monotherapy for treatment-naïve WHO FC II-III patients and as add-on therapy in WHO FC III-IV patients currently on stable doses of ERAs or inhaled prostanoids [10]. The recently updated ESC/ERS guidelines recommend riociguat as monotherapy in WHO FC II-III patients (Grade IB) and as sequential drug combination therapy with bosentan in WHO FC II-III patients (Grade IB) [7].

The phase III PATENT-1 trial of riociguat allowed PAH patients on stable background ERA therapy who remained symptomatic to receive individualized doses of riociguat up to a maximum of 2.5 mg TID [11]. Results showed a mean placebo-corrected change in in 6MWD from baseline to last visit of 36 m. Further, the mean change in PVR was −174 ± 202 dyn∙s∙cm^−5^ for riociguat versus −46 ± 266 dyn∙s∙cm^−5^ for placebo. By last visit, 26 % of riociguat patients had improved one WHO-FC (71 % maintained FC, while 4 % worsened 1 FC) compared with 13 % of placebo patients (76 % maintained FC, and 9 % and 2 % worsened 1 and 2 FC, respectively) [11].

Administration of riociguat is contraindicated in patients currently receiving PDE-5i’s due to the risk of hypotension [12]. Until recently, treatment paradigms were sequential and additive, with PDE-5i’s traditionally first-line add-on therapy, and ongoing studies are investigating the efficacy of riociguat combination therapy in patients with insufficient response to PDE-5i’s. The RESPITE trial is an open-label, single-arm, uncontrolled phase 3b study investigating whether patients who do not have an adequate response to therapy with PDE-5i’s may have insufficient synthesis of cyclic guanosine monophosphate (cGMP), and as such may respond to sGC stimulators [13]. PAH patients with an insufficient response to stable doses of sildenafil or tadalafil for at least 90 days will be switched to riociguat for 24 weeks. Patients pretreated with a stable dose of sildenafil for ≥90 days will be switched to riociguat after a 24-h washout and those pretreated with a stable dose of tadalafil for ≥90 days will be switched to riociguat after a 72-hourwashout. Riociguat will be individually dose-adjusted in 0.5-mg increments during an 8-week titration phase to a maximum of 2.5 mg TID. A 16-week maintenance phase will assess change from baseline in 6-min walk distance at 24 weeks as well as changes in cardiac index, NT-proBNP, WHO FC, quality of life, and the proportion of patients with clinical worsening. All endpoints in the study are exploratory [13]. Results from the phase 3b RESPITE study will determine if PAH patients who have an insufficient response to PDE-5i therapy are likely to respond to treatment with an sGC stimulator. The sGC stimulator riociguat is the first drug in its class to be approved, and as it does not require the presence of NO to stimulate sGC, riociguat may provide benefits for patients who do not respond to PDE-5i due to NO deficiency [14].

## Conclusion

PAH is a life-threatening disease that requires vigilance in therapeutic management to ensure that patients remain stable for as long as possible. Treatment of PAH is complicated not only by the multifactorial nature of the disease but also by larger issues around medication access. As new PAH agents are developed, clinicians must decide how to integrate them into the treatment algorithm. In the absence of formal guidelines for switching stable patients to the novel PAH agent riociguat, this case offers evidence of the potential for a safe and clinically beneficial transition from a first-line PDE-5i. In our patient the transition from PDE-5i to riociguat was performed without washout. The safety of this approach with the longer acting PDE-5i, tadalafil, is unknown. Similarly, transition without washout should be approached cautiously in patients with low baseline systemic blood pressure.

### Consent

Written informed consent was obtained from the patient for publication of this case report and any accompanying images. A copy of the written consent is available for review by the Editor of this journal.

### Availability of data and materials

The data supporting the conclusions of this article are available upon request to the corresponding author.

## Abbreviations

6MWD: six-minute walk distance
BID: twice daily
cGMP: cyclic guanosine monophosphate
CI: cardiac index
CO: cardiac output
CTEPH: chronic thromboembolic pulmonary hypertension
DOE: dyspnea on exertion
ERA: endothelin receptor antagonist
ERS: European Respiratory Society
ESC: European Society of Cardiology
FC: functional class
mPAP: mean pulmonary arterial pressure
NO: nitric oxide
NYHA: New York Heart Association
PAH: pulmonary arterial hypertension
PDE-5i: phosphodiesterase-5 inhibitor
PO: oral
PVR: pulmonary vascular resistance
RHC: right heart catheterization
sGC: soluble guanylate cyclase
TID: three times daily
V/Q: ventilation-perfusion scan
WHO: World Health Organization
